# Building flux capacity: Citizen scientists increase resolution of soil greenhouse gas fluxes

**DOI:** 10.1371/journal.pone.0198997

**Published:** 2018-07-05

**Authors:** Cody C. Reed, Julianne M. Winters, Stephen C. Hart, Rachel Hutchinson, Mark Chandler, Gitte Venicx, Benjamin W. Sullivan

**Affiliations:** 1 Department of Natural Resources & Environmental Science, The University of Nevada Reno, Reno, Nevada, United States of America; 2 Department of Integrative Biology, University of California Berkeley, Berkeley, California, United States of America; 3 Life & Environmental Sciences and Sierra Nevada Research Institute, University of California Merced, Merced, California, United States of America; 4 South Yuba River Citizens League, Nevada City, California, United States of America; 5 Earthwatch Institute, Allston, Massachusetts, United States of America; 6 The Global Water Center, The University of Nevada Reno, Reno, Nevada, United States of America; The University of Sydney, AUSTRALIA

## Abstract

Though citizen science programs have been broadly successful in diverse scientific fields, their adoption has lagged in some disciplines, including soil science and ecosystem ecology. Collaborations with citizen scientists may be viewed as a conundrum in these disciplines, which often require substantial labor and technical experience; citizen scientists could improve sampling capacity but may reduce sample quality or require training and oversight prior to and while performing specialized tasks. To demonstrate the feasibility of incorporating citizen scientists into soil biogeochemistry research, we conducted a proof-of-concept study in high-elevation meadows of the Sierra Nevada in California. A collaboration between university researchers and citizen scientists allowed us to assess spatial and diel patterns of soil greenhouse gas (GHG) fluxes with an intensity and frequency that would otherwise be beyond the capacity of a typical research laboratory. This collaboration with citizen scientists increased our sampling intensity by over 700% while only doubling the sampling error relative to that of full-time researchers. With training and support from project scientists, citizen scientists collected data that demonstrate spatial independence of carbon dioxide, methane, and nitrous oxide at scales between 1 m and 175 m. Additionally, we found a lack of temporal variation over a 24-h period for all three GHGs. Citizen scientists participating in this one-day event reported levels of satisfaction commensurate with longer-term, immersive campaigns. The place-based event also proved an effective tool for teaching intangible concepts of soil biogeochemistry and promoting local conservation. Despite perceived barriers to entry, this study demonstrates the mutual benefits of citizen science collaborations in soil science and ecosystem ecology, encouraging adoption by disciplines that have been slow to take advantage of such collaborations. Short-term, local citizen science events can provide meaningful experiences for area residents and teach global biogeochemical cycles in a place-based context.

## Introduction

Citizen science is recognized as a valuable approach for achieving scientific research objectives and promoting public participation and interest in science [1]. In ecological research, citizen science programs are well regarded for their ability to engage volunteers in participatory experiences that promote place-based learning by educating participants through experiences with local ecological properties and processes. Additionally, they foster scientific inquiry while allowing scientists to ask questions at ambitiously broad scales [2, 3]. Popular citizen science programs address biogeographic or demographic questions associated with flora and fauna (e.g., iNaturalist, eBird, National Phenology Network) or other natural phenomena (e.g., Aurorasaurus, Space Weather). In these programs, networks of geographically dispersed citizen scientists permit regional or continental scale monitoring, and the development of large, otherwise unattainable, datasets [2, 3, 4].

Despite these examples of successful collaborations, other disciplines, including soil science and ecosystem ecology, have been slower to adopt citizen science programs [5, 6]. This latent adoption may be attributed to numerous perceived barriers to entry for ecologists and citizen scientists alike. Such barriers could include, but are not limited to, the need for training on specialized sampling techniques or equipment, and the cost and availability of sample processing. However, collaborations with citizen scientists could allow researchers to increase their scope of inquiry and better capture the spatial and temporal variability of ecosystem-scale pools and processes. Simultaneously, hands-on experiences could be used to teach abstract concepts of soil biogeochemistry to the public in a tangible manner. A proof-of-concept study could expand citizen science programs in this field by demonstrating feasibility and success for citizen scientists and researchers alike.

Meadows in the Sierra Nevada of California offer an ideal location for a proof-of-concept collaboration between citizen scientists and soil biogeochemists due to their proximity to large population centers, aesthetic appeal, and ecological relevance. High-elevation Sierra meadows supply water to over 25 million people [7] and are biodiversity hotspots that contain high densities of soil carbon (C) [8]. Meadow ecosystems are reliant on hydrologically connected floodplains with shallow groundwater tables that fluctuate as a result of seasonal changes, interannual climate variability, and anthropogenic disturbances. This dynamic hydrology creates hotspots of anaerobic and aerobic biogeochemical processes which—depending on conditions—could make meadow soils important sinks or sources of greenhouse gases (GHGs) such as carbon dioxide (CO_2_), methane (CH_4_), and nitrous oxide (N_2_O). Beyond the effects of hydrology, the direction and magnitude of these fluxes are governed by abiotic and biotic factors (e.g., temperature, soil chemistry, microbial activity, and plant productivity) that can vary daily, seasonally, and spatially from molecular to landscape scales [9].

Presently, the extent to which Sierra meadows are net sources or sinks of GHGs remains unresolved [10]. This lack of knowledge presents a barrier to restoration strategies designed to increase C sequestration or mitigate GHG fluxes [11]. Robust annual estimates of GHG fluxes from Sierra Nevada meadows will require quantification of spatial and diel variation prior to designing long-term monitoring efforts in order to ensure proper spatial and temporal sampling intensity. Such measurements are resource-intensive and often beyond the capacity of a single investigator because of the need for monitoring across fine spatial and temporal scales. This scientific need represented an opportunity to test whether collaboration with citizen scientists could increase sampling capacity to improve spatial and temporal resolution of GHG fluxes in meadows, while providing valuable educational experiences for local volunteers.

Our study had two objectives. We sought to: (1) evaluate whether citizen science could be successfully incorporated into soil biogeochemistry research, and (2) assess the feasibility of using citizen scientists to measure spatial and diel dynamics of GHG fluxes in Sierra Nevada meadow ecosystems. For the first objective, we expected that citizen scientists, trained by soil biogeochemists, could collect samples with low sampling error, and that this experience would be positive and enhance their scientific knowledge. For the second objective, we hypothesized that GHG fluxes from meadow soil would be sensitive to diel variation in temperature and vary across the landscape with spatial autocorrelation at the meter scale. To address these objectives, we partnered with citizen scientists to conduct a 24-h research campaign designed to maximize data quality and scientific engagement, while measuring GHG fluxes at high temporal and spatial resolution. The results of this study provide a successful proof-of-concept for the integration of citizen scientists into ecosystem ecology research.

## Methods

This study was conducted on August 29–30, 2015 in Loney Meadow, a 19-ha riparian low gradient meadow [12] that sustains one perennial and many seasonal streams. Loney Meadow is located at 1800 m elevation in the Tahoe National Forest on the western slope of the Sierra Nevada (39° 25' 15.3113'' N, 120° 39' 17.5831'' W). The meadow was under private ownership until 1989 and has a history of intensive grazing, logging and mining dating back to the late-1800s. The intensity of the grazing has steadily decreased since the 1960s but the site was still actively grazed at the time of the study as part of a US Forest Service grazing allotment. The area is characterized by a Mediterranean climate, with a mean annual air temperature of 9.4°C and a mean annual precipitation of 1600 mm [13]. No permits were required as only non-invasive sampling methods were used and no vegetation or soil samples were taken.

For the campaign, we recruited 15 citizen scientists from the local area. Citizen outreach was performed by a regional not-for-profit restoration and conservation organization, the South Yuba River Citizens League (SYRCL) in collaboration with Earthwatch Institute. Earthwatch Institute is an environmental non-profit which recruits over 2000 citizen scientists each year to participate and contribute to over 60 scientific research endeavors worldwide. The participants ranged in age from 15 to 68 and included high school students, an elementary school teacher, retirees, and an emergency medicine physician’s assistant, among others. The participants were trained on site and supported throughout the campaign by 4 researchers: 2 university professors and 2 graduate students. Training lasted approximately 1 hour and included a demonstration of and hands-on experience with the static chamber method of GHG sampling (details below). Researchers also introduced participants to the meadow C cycle and basic mechanisms underlying ecosystem GHG fluxes. The duration of individual citizen scientist participation ranged from 5 to 24 hours. Scientific results were shared with participants during a webinar hosted by Earthwatch in December 2015 (webinar archived at: https://www.youtube.com/watch?v=jaaafkPy-Wk&feature=youtu.be).

Written surveys of participant experiences were conducted after the campaign was complete. The survey was similar to those used in standard research campaigns run by the Earthwatch Institute. This similarity allowed us to measure whether participants experienced benefits from this short-term, local campaign comparable with those obtained from longer-term, often international, trips commonly offered by Earthwatch. Slight modifications were made only to tailor the questions to the research objectives. The survey consisted of 4 Likert scale and 4 short answer questions related to participant experience and the impact of the event on their scientific understanding and views of the natural world (S1 File)[14]. Surveys took participants less than 10 minutes to complete. Following consultation with the Director of Research Integrity at the University of Nevada Reno, we determined that this study was exempt from review by the Institutional Review Board because citizen scientists participated voluntarily with full knowledge of the study design. Surveys given to citizen scientists were anonymous and contained no identifying information. Identifying characteristics reported were obtained through voluntary personal communications.

We sampled soil GHG fluxes using vented static chambers as described in Hutchinson and Mosier [15]. Chambers were constructed of vented polyvinyl chloride (PVC) sewer end caps fitted with rubber septum and vent tubes. Rubber gaskets inside the chambers allowed them to be secured to PVC collars 20 cm in diameter. Collars were approximately 11.25 cm tall and were inserted approximately 2 cm into the soil at least one hour prior to sampling. Instructions we used for static chamber construction are available at dx.doi.org/10.17504/protocols.io.nntdden. Chambers were secured to the collar immediately before sampling. Headspace gas samples were collected 0, 15, and 30 minutes after the chamber tops were secured and 17 mL of headspace stored in 12 mL evacuated Exetainers (Labco Limited, Lampeter, U.K.). Detailed field sampling methods are available at dx.doi.org/10.17504/protocols.io.nnsddee. Gas samples were transported to the lab and analyzed for CO_2_, CH_4_, and N_2_O concentrations using a gas chromatograph (GC-2014, Shimadzu Scientific Instruments, Columbia, MD, USA). During each sampling period, air temperature and barometric pressure were measured using a handheld barometer and these measurements later used to calculate the moles of GHG present in the chamber headspace at each sampling period based on the ideal gas law. Within 0.5 m of each static chamber, we measured soil temperature using an analog thermometer and volumetric water content using time domain reflectometry (TDR; FieldScout 100, Spectrum Technologies, Aurora, IL, USA) to a depth of 7.5 cm.

In order to test the spatial independence of GHG flux measurements, we began with a 24-point, 60 x 150 m grid with 30 m between collars. The grid was located in a representative area that would not be directly disturbed by future meadow restoration activities. Centered around four points, we superimposed a second grid of 28 collars at distances of 1.5, 3, 5, and 10 m from the original collar locations. By combining these two grids and working with the team of 15 citizen scientists, we were able to sample all 52 collars simultaneously and measure the spatial independence of fluxes from 1.5–175 m. We assessed relative spatial autocorrelation of fluxes from these 52 collars by plotting semivariance values for all paired distances in a semivariogram using the ‘gstat’ package in R [16].

To measure diel variability of GHG fluxes, we sampled 18 collars from the original 30 m grid every two hours over a 24-h period. We compared temporal differences in mean GHG fluxes using a repeated measures analysis of variance (RMANOVA). The predictive value of environmental variables (air temperature, soil temperature, and volumetric water content) on both spatial and temporal variation was tested using linear regression.

## Results

### Objective 1: Citizen scientists as soil biogeochemists

**Fig 1:**
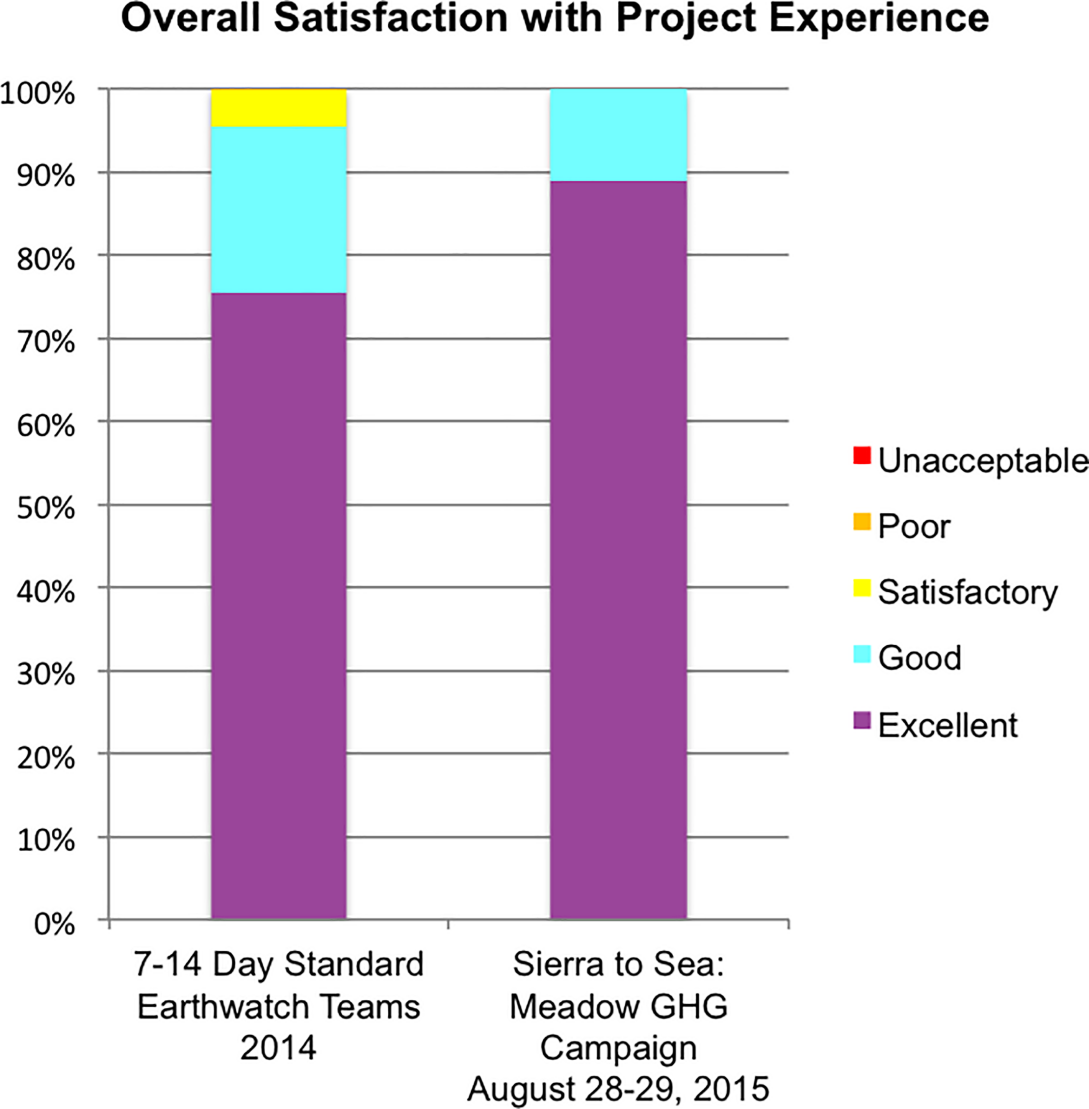
Participant experience compared to that of Standard Earthwatch Teams. Participants in the ‘local’, 1-day Sierra to Sea: Meadow greenhouse gas (GHG) research campaign also reported higher levels of overall satisfaction than those achieved after ‘exotic’ 7–14 day Standard Earthwatch Teams (N = 911 participants, 72 campaigns).

Surveys completed by citizen scientists indicated the campaign was both a positive and educational experience (60% response rate; Table 1). The majority reported that participation in this one-day campaign increased their conceptual understanding of the importance of GHG research and value of meadow ecosystems. They also stated that participation enhanced their connection with the natural world and motivated them to take action towards a sustainable environment. All participants reported that the campaign significantly increased their understanding of the general contribution of citizen science and their interest in participating in future citizen science events. Surveys completed following this campaign also compared favorably with those from long-standing Earthwatch campaigns. One hundred percent of citizen scientists surveyed reported their overall satisfaction with the experience as either “good” or “excellent”—the highest two levels of response—compared with 95% of volunteers participating in standard 7–14 day Earthwatch Institute research campaigns in 2014 (Fig 1).

**Table 1:** Citizen science survey results.

Participation in Sierra to Sea: Meadow GHG campaign increased volunteer:	% responding at highest 2 levels of response	Average score (range of 0–5	Standard deviation	N
Understanding of importance of measuring GHG fluxes and relationship of research to global environmental issues	89%	4.89	0.33	9
Understanding of the value of meadow habitats in combating climate change	89%	4.22	0.66	9
Personal connection to the natural world	88%	4.25	0.71	8
Commitment to take positive action toward environmental sustainability	88%	4.25	0.71	8
Motivation to include environmental considerations in day-to-day decision making	50%	3.63	0.74	8
Understanding of contribution of CS and interest in CS events	100%	4.75	0.46	8

Results from surveys conducted following Earthwatch citizen science campaigns. Citizen science (CS) participants reported that involvement in this campaign increased their understanding of environmental issues and commitment to action toward environmental sustainability.

Incorporating citizen scientists into the campaign permitted an increase in the spatial intensity of GHG flux sampling of 766% and a nine-fold increase in the number of fluxes collected over a 24-h period, compared with the number typically collected by a single researcher. This translated to an 11-fold increase over a typical sampling event in the amount of time each sampling point was measured during a day (Table 2). In a typical sampling event, a static chamber covers each collar for 30 minutes per day. During this campaign, the 18 collars used for diel sampling were covered for 360 minutes. The inclusion of newly trained citizen scientists did result in an increase in sampling error (defined as the number of samples excluded from flux calculations due to possible sampling error; citizen scientists averaged 2.7% vs. 0.7% for trained researchers) but remained well below our target goal of 5%. Additionally, the percent error was higher during the spatial sampling conducted immediately after training (3.9%) than during the diel sampling (2.1%). This may be explained by the fact that during the diel sampling each participant was involved with multiple measurements. Causes of sampling error included, but were not limited to, a poor seal between the chamber and the soil leading to a flat CO_2_ flux, unfilled Exetainers, or mislabeled samples.

**Table 2:** Citizen scientists increased sampling resolution with only a small increase in sampling error.

Metric	Typical day	With citizen scientists	Increase (%)
Number of fluxes collected	24	244	916
Collars sampled simultaneously	6	52	766
Number of static chambers needed	6	52	766
Number of minutes greenhouse gases captured (per 24-h period)	30	360	1100
Sampling error (%)	0.72	3.9 (spatial)	441
		2.1 (temporal)	191

Increase in sample intensity and error as a result of incorporating citizen scientists into meadow greenhouse gas field sampling, compared with that achieved by a single, trained researcher. Increase is reported as the percent difference in the metric with and without citizen scientists.

### Objective 2: Spatial and diel dynamics of GHG fluxes

**Fig 2:**
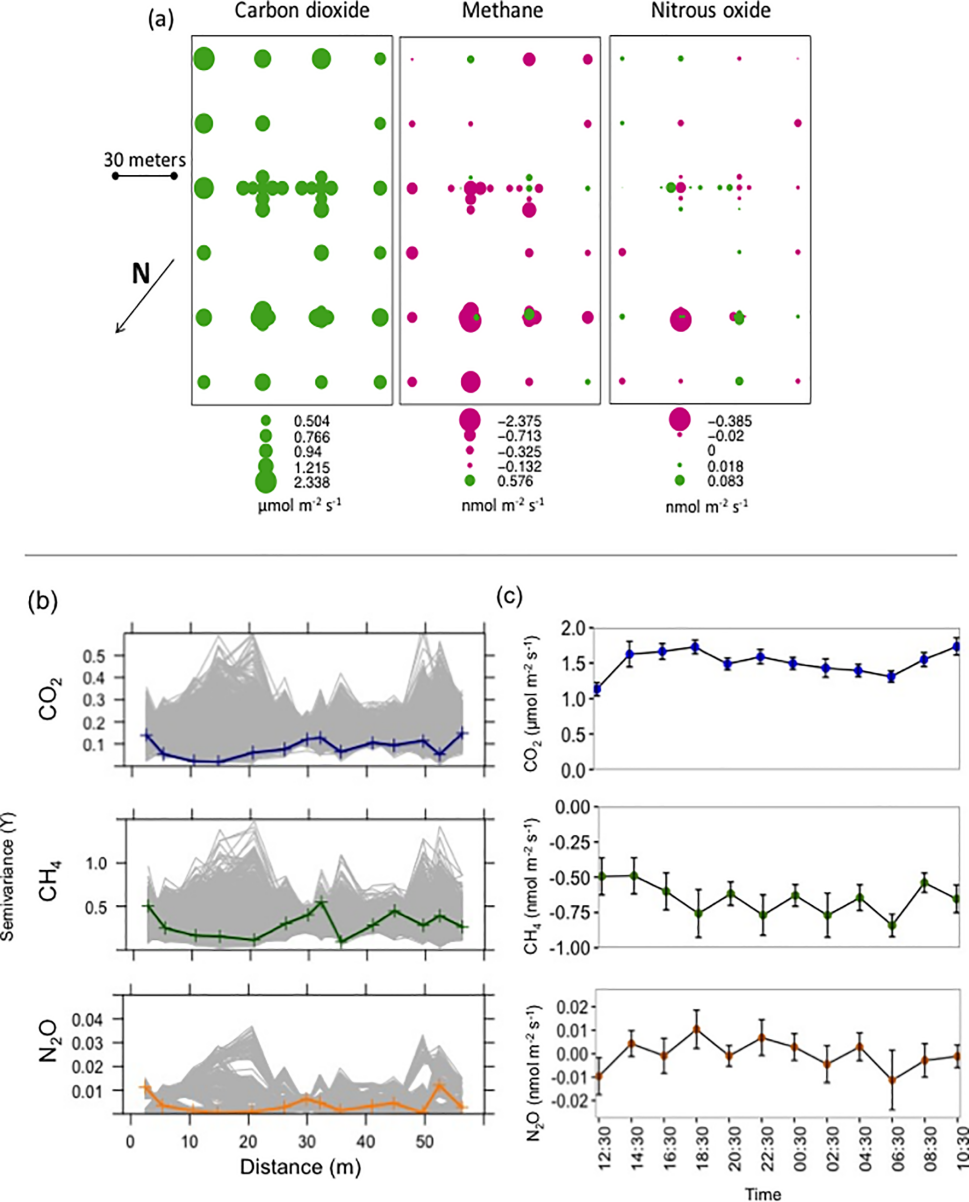
Spatial and temporal variation of greenhouse gas fluxes. (A) Spatial variation of carbon dioxide (CO_2_), methane (CH_4_), and nitrous oxide (N_2_O) fluxes. Bubble size corresponds with flux size. Green bubbles indicate positive and pink negative fluxes. (B) Sample semivariograms (colored line) do not differ from 1000 semivariograms randomly resampled from the same data (gray lines). Lack of change in semivariances with distance suggests greenhouse gas fluxes were spatially independent of each other at scales greater than 1.5 m. (C) Flux values for CO_2_, CH_4_, and N_2_O sampled every two hours over a 24-h period. No significant diel variation was observed. Data are mean ± 1 standard error.

**Fig 3:**
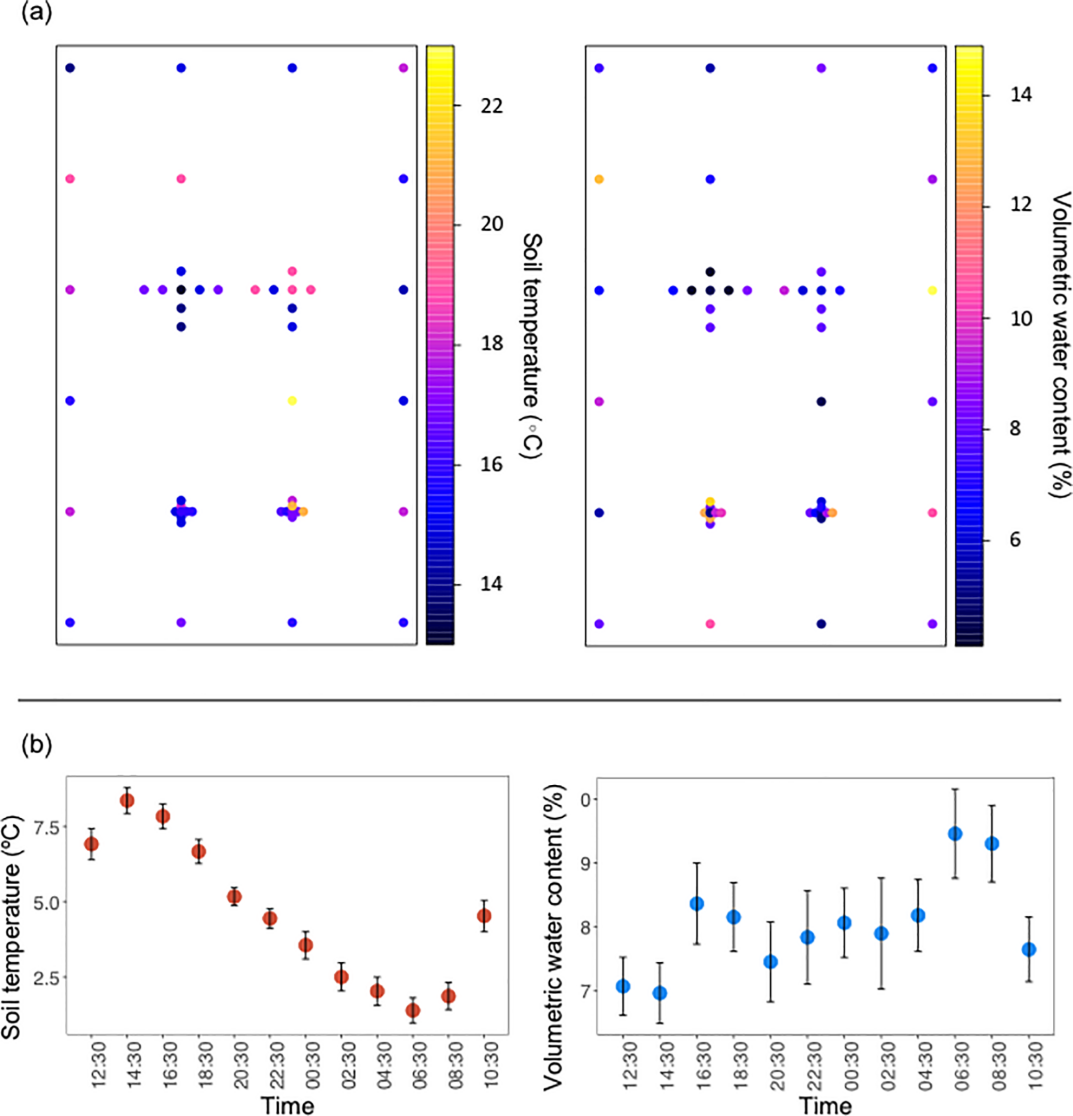
Spatial and temporal variation of soil moisture and temperature. (A) Spatial variation of volumetric water content and soil temperature. Bubble color corresponds with value. Higher values are warmer colors. (B) Soil moisture and temperature values sampled every two hours for 24 hours. Significant diel variation was observed for soil temperature but not volumetric water content. Data are means ± 1 standard error.

Fluxes of all three GHGs (CO_2_, CH_4_, and N_2_O) exhibited high spatial variability but did not display clear spatial patterns within the meadow (Fig 2A). Mean CO_2_ flux during the spatial sampling period was 1.05 ± 0.05 μmol m^-2^ s^-1^, with a coefficient of variation of 37%. The mean CH_4_ flux was negative (-0.44 ± 0.08 nmol m^-2^ s^-1^, CV = 133%), indicating that, on average, CH_4_ uptake exceeded CH_4_ production (although 10 of the 52 collars sampled showed net CH_4_ production). We measured both uptake and emission of N_2_O, but mean fluxes were generally the lowest of the three GHGs measured (mean: -0.008 ± 0.008 nmol m^-2^ s^-1^, CV = 788%). Additionally, sample semivariograms for all three gases did not differ from 1000 semivariograms from randomly reallocated data, suggesting a lack of spatial autocorrelation among sample points [17]. Unexplained variance (nugget variance) was high relative to sample variance, providing further evidence that GHG fluxes lacked spatial autocorrelation on the scales measured (1.5 to 175 m; Fig 2B). Soil volumetric water content did not vary with distance to stream channel and showed a similar lack of spatial patterning with a mean of 7.96% and a coefficient of variation of 33% (Fig 3A). A significant but weak negative relationship was found between volumetric water content and all three GHGs (CO_2_: F = 4.08, *p* = 0.05, r^2^ = 0.07; CH_4_: F = 4.9, *p* = 0.03, *r*^*2*^ = 0.08; N_2_O: F = 5.1, *p* = 0.03, r^2^ = 0.09). No significant relationships existed between GHG fluxes and soil temperature during the spatial sampling (CO_2_: F = 0.50, *p* = 0.49; CH_4_: F = 1.25, *p* = 0.27; N_2_O: F = 0.91, *p* = 0.35).

During the diel sampling, no significant variation in mean flux was found for any of the three gases over the 24-h period (CO_2_: *F* = 1.31, *p* = 0.22; CH_4_: *F* = 0.56, *p* = 0.86; N_2_O: F = 0.93, *p =* 0.512, N = 18; Fic 2C). Mean fluxes were also similar to those measured during the spatial sampling (CO_2_: 1.52 ± 0.03 μmol m^-2^ s^-1^; CH_4_: -0.65 ± 0.03 nmol m^-2^ s^-1^ N_2_O: -0.0002 ± 0.002 nmol m^-2^ s^-1^). Variation in mean CH_4_ flux during diel sampling was best explained by changes in air temperature (F = 13.4, *p* < 0.005, r^2^ = 0.53). Soil temperature varied significantly over the 24-h period (F = 8.93, *p* < 0.005), but no significant variation was measured for volumetric water content (F = 0.97, *p* = 0.48; Fig 3B). No significant relationships existed between soil volumetric water content or soil temperature and mean GHG fluxes (VWC: CO_2_: F < 0.001, *p* = 0.99; CH_4_: F = 2.44, *p* = 0.15; N_2_O: F = 0.70, *p* = 0.42. Soil Temperature: CO_2_: F = 1.09, *p* = 0.32; CH_4_: F = 3.09, *p =* 0.11; N_2_O: F = 1.15, *p* = 0.31).

## Discussion

This proof-of-concept study demonstrated that incorporation of citizen scientists into soil biogeochemical research can be beneficial for volunteers and scientists alike. Survey results from citizen scientists indicated the experience was positive and educational for participants. Incorporation of citizen scientists into the field-based campaign allowed researchers to achieve high resolution GHG sampling with an acceptable increase in sampling error.

Citizen scientists participating in this study reported levels of satisfaction equivalent to those achieved after weeklong field expeditions using similar evaluation tools. This suggests that participation in local and short duration research can be equally impactful as participation in longer-term programs. Our results also support findings that place-based citizen science events facilitate tangible learning of intangible ecosystem function and promote local conservation efforts [18, 19]. The proximity of our study site to the communities where the volunteers live allowed us to introduce global environmental issues in a local context, helping individuals gain appreciation for meadow ecosystems and understand the impact of the C and hydrologic cycles in their everyday lives (personal observation). The weekend-long format facilitated involvement by high school students and working professionals, thereby increasing the diversity of participants and scope of impact.

Collaborating with citizen scientists allowed us to determine that GHG fluxes in this meadow exhibited little spatial autocorrelation at distances >1m along with no significant diel variation. This was contrary to our expectations based on the fact that Sierra meadows often have clear hydrologic, vegetative, and edaphic gradients. Previous studies have found spatial dependence of CO_2_, CH_4_, and N_2_O at distances less than 50–100 m in drained peatlands [20] and less than 10 m for CH_4_ in forest soil [21] and N_2_O in mowed grasslands [22]. However, a lack of spatial autocorrelation of GHG fluxes has been measured at scales greater than 1 m in grazed grasslands and other human-managed landscapes [22, 23]. Active grazing in this meadow may have increased site heterogeneity and contributed to the absence of observed spatial autocorrelation at distances > 1m. As such, our results suggest that GHG fluxes in this grazed meadow ecosystem were regulated by biogeochemical or biophysical processes occurring at sub-meter scales.

Despite the location of the grid alongside the stream channel, the absence of clear spatial patterns of soil moisture may be a function of low groundwater levels during the season when we sampled. Groundwater levels were lower than the deepest point in the stream, effectively disconnecting the floodplain and removing the influence of the stream channel. Such conditions are common in degraded meadows throughout the Sierra Nevada, especially during the growing season when the combination of evapotranspiration, losing stream reaches, and lack of precipitation often lowers water tables below the bottom of the stream channel [24]. Under these conditions, microtopography may have greater influence on soil water than proximity to the channel. Similarly, the weak correlation of soil moisture with GHG fluxes may be explained by low spatial variation in soil moisture. Consequently, these results do not imply that there is no relationship between GHG fluxes and environmental variables throughout the year, but only that they were decoupled during this fall sampling event.

We expected to measure significant diel variation in soil GHG fluxes during our 24-h sampling campaign because of large air temperature changes in late summer at high elevations. We predicted fluxes would be lower at night, when air temperatures decreased from 20°C to 4°C and soil temperatures decreased from 18°C to 12°C, because temperature should be a first-order physical control over soil microbial and enzyme activity [9]. Despite this theoretic basis and despite significant changes in soil temperature during the 24-h period, we found no significant diel variation in GHG fluxes. A lack of diel variability in GHG fluxes has been previously found in several other studies in diverse ecosystems [20, 25, 26, 27], but to our knowledge this represents the first such finding in meadow soils.

Both the spatial and diel flux measurements obtained with the assistance of citizen scientists have important implications for scaling future meadow GHG measurements across space and time. For instance, GHG fluxes sampled at scales > 1 m in human-managed ecosystems are likely spatially independent, suggesting sub-meter measurements may not be needed to identify GHG emission “hot spots” [28], or for extrapolating GHG fluxes from chamber to meadow spatial scales. Additionally, the absence of diel variability suggests daytime GHG flux measurements may be extrapolated over a 24-h period without bias when developing annual GHG budgets, thereby simplifying the number of measurements required and reducing the overall cost of the measurement campaigns.

We recognize these results present only a glimpse of GHG fluxes in Sierra Nevada meadow ecosystems and caution against extrapolation of our results across seasons or ecosystems. Repeated sampling under a variety of conditions will be required to fundamentally increase our understanding of the spatial and temporal variability of GHG fluxes in meadows. Nevertheless, this case study demonstrates that citizen scientists, working alongside project researchers, can make valuable contributions to GHG research.

Incorporating citizen scientists increased our sampling intensity by over 700% and allowed us to addressing otherwise infeasible research questions. While the rate of sampling error did go up with citizen scientists (3.9% and 2.1% during the spatial and temporal samplings compared with 0.7% by trained researchers), the increase was acceptable given the additional temporal and spatial resolution achieved. Sampling error decreased during the diel sampling after participants had sampled multiple times, indicating that sampling error associated with more experienced volunteers may approach error rates of full-time research personnel. However, we posit that the hands-on training of campaign volunteers by research personnel contributed greatly to the reliability of the results. This format also facilitated informal scientific discussion and contributed to positive educational and conservation outcomes.

While the overall impact was positive, the inclusion of citizen scientists created novel challenges and limitations. Almost an eight-fold increase in field supplies required substantial additional time for organization and preparation. Researchers were also required to be flexible and creative to ensure reliability of results, while dealing with unexpected circumstances that arose with volunteers. While not a factor in this study, inflexibility once campaign dates are set may preclude incorporation of citizen scientists with research that is sensitive to weather or timing of biological events. The close involvement of research personnel also helped us avoid issues of data fragmentation and inaccuracy noted by other studies using citizen scientists [29].

Ultimately, the success of this case study stemmed from the efforts of an interdisciplinary team that placed equal weight on research and educational objectives using a deliberate design process [30, 6]. Despite the coordination required for such an approach, the educational, outreach and scientific benefits greatly offset the additional effort. Our study also demonstrates that short-duration, place-based citizen science campaigns can be effective tools for promoting personal connection with valuable ecosystems and motivating local conservation efforts.

## Supporting information

S1 File Citizen science survey. (PDF)

S2 File Citizen science survey results. (XLSX)

S3 File Greenhouse gas flux data for spatial sampling. (XLSX)

S4 File Greenhouse gas flux data for temporal samplings. (XLSX)
