# Supplementary material for: Building flux capacity: Citizen scientists increase resolution of soil greenhouse gas fluxes
Source: PLoS One. 2018 Jul 5;13(7):e0198997. doi: 10.1371/journal.pone.0198997 (PMC6033390; doi:10.1371/journal.pone.0198997)
Supplement: S1 File — (PDF) [file pone.0198997.s001.pdf]

Sierra to Sea Evaluation Form

Please take time to complete the evaluation form below and return it to your Team Leader. Your comments will help us to monitor and improve the program. We value your suggestions!

General Information

**Name** (optional)				
**Team Leader**		**Meadow Name**		
**Date**		**May we quote you?**	Yes	No

Evaluation

	**Unacceptable**	**Poor**	**Satisfactory**	**Good**	**Excellent**
**Field Experience**					
**1. Please rate the following elements of your experience:**					
a) quality of prearation matierals					
b) rendezvous and welcome	1	2	3	4	5
c) logistical arrangements (organization, scheduling etc.)	1	2	3	4	5
d) health & safety briefings delivered by project staff	1	2	3	4	5
e) training on field research tasks	1	2	3	4	5
f) sense of involvement in the research	1	2	3	4	5
g) explanation of relevance & significance of research	1	2	3	4	5
h) overall support & leadership from the project staff	1	2	3	4	5
i) overall satisfaction with project experience	1	2	3	4	5

	**Not at all**	**Minimally**	**Moderately**	**Significantly**	**Very significantly**
**Research & Sustainability**					
**2. To what extent did participation on the project increase your understanding of:**					
a) the environmental issues being addressed	1	2	3	4	5
b) how the research relates to global environmental issues	1	2	3	4	5
c) how the research is relevant to the local communities in which it is conducted	1	2	3	4	5
d) why we need to collect data on greenhouse gas flux	1	2	3	4	5
e) the value of meadow habitat to combatting climate change	1	2	3	4	5
f) how the research will be used	1	2	3	4	5
g) the general contributions of citizen science	1	2	3	4	5

	Not at all	Minimally	Moderately	Significantly	Very significantly
**3. To what extent did participation on the project:**					
a) provide an opportunity for you to make a meaningful contribution to improving the state of the planet	1	2	3	4	5
b) increase your sense of personal connection to the natural world	1	2	3	4	5
c) increase your confidence in your ability to make a difference towards a sustainable environment or community	1	2	3	4	5
d) increase your commitment to take positive action towards a sustainable environment or community	1	2	3	4	5
e) motivate you to include more environmental considerations in your day-to-day decision making	1	2	3	4	5
**4. Please rate how your participation has:**					
a) increased your likelihood to recommend Sierra to Sea to others	1	2	3	4	5
b) increased your commitment to citizen science	1	2	3	4	5

**Program Feedback**
**5. What did you particularly enjoy about this program?**
**6. Have you participated in citizen science before today? Would you do it again?**
**7. What do you hope to bring back to your daily life in terms of environmental consiousness, community outreach, or new knowledge from this experience?**
**8. What could have been done to improve your experience?**

Thank you for taking the time to complete this evaluation. Your assistance is much appreciated
